# Evaluation of a self-imaging SD-OCT system designed for remote home monitoring

**DOI:** 10.1186/s12886-022-02458-z

**Published:** 2022-06-10

**Authors:** Judy E. Kim, Oren Tomkins-Netzer, Michael J. Elman, David R. Lally, Michaella Goldstein, Dafna Goldenberg, Shiri Shulman, Gidi Benyamini, Anat Loewenstein

**Affiliations:** 1 The Eye Institute, Medical College of Wisconsin, 925 N. 87th street, Milwaukee, WI 53226 USA; 2 Department of Ophthalmology, Lady Davis Carmel Medical Center, Ruth and Bruce Rappaport Faculty of Medicine, Technion, Haifa, Israel; 3 Elman Retina Group PA, Baltimore, MD USA; 4 Retina Research Institute at New England Retina Consultants, Springfield, MA USA; 5 Tel Aviv Medical Center, Tel Aviv, Israel; 6 Assuta Medical Centers, Tel Aviv, Israel; 7 Notal Vision, Tel Aviv, Israel

**Keywords:** Age-related macular degeneration, HOME OCT, Home monitoring

## Abstract

**Purpose:**

To compare identification rates of retinal fluid of the Notal Vision Home Optical Coherence Tomography (OCT) device (NVHO) when used by people with age-related macular degeneration (AMD) to those captured by a commercial OCT.

**Methods:**

Prospective, cross-sectional study where patients underwent commercial OCT imaging followed by self-imaging with either the NVHO 2.5 or the NVHO 3 in clinic setting. Outcomes included patients’ ability to acquire analyzable OCT images with the NVHO and to compare those with commercial images.

**Results:**

Successful images were acquired with the NVHO 2.5 in 469/531 eyes (88%) in 264/290 subjects (91%) with the mean (SD) age of 78.8 (8.8); 153 (58%) were female with median visual acuity (VA) of 20/40. In the NVHO 3 cohort, 69 eyes of 45 subjects (93%) completed the self-imaging. Higher rates of successful imaging were found in eyes with VA ≥ 20/320. Positive percent agreement/negative percent agreement for detecting the presence of subretinal and/or intraretinal fluid when reviewing for fluid in three repeated volume scans were 97%/95%, respectively for the NVHO v3.

**Conclusion:**

Self-testing with the NVHO can produce high quality images suitable for fluid identification by human graders, suggesting the device may be able to complement standard-of-care clinical assessments and treatments.

**Supplementary Information:**

The online version contains supplementary material available at 10.1186/s12886-022-02458-z.

## Introduction

Most vision loss associated with age-related macular degeneration (AMD) is caused by atrophy or neovascularization with exudative changes occurring at the macula [1]. While neovascular AMD (nAMD) represents only 10–15% of all AMD, it is responsible for more than 80% of all AMD-related vision loss [2]. Several treatment regimens exist for the management of eyes with nAMD using intravitreal anti-vascular endothelial growth factor (VEGF) injections, including injections performed monthly [3], treat-and-extend [4, 5] and pro re nata (PRN) [6, 7]. In addition to visual acuity (VA) and clinical findings based on fundus examinations, the assessment of the macula and the decision to treat or not are mainly driven by interpretation of retinal scans from optical coherence tomography (OCT) systems performed in the office during the patient visit. Spectral domain OCT (SD-OCT) is currently most commonly used and has excellent capabilities to image intraretinal, subretinal, and subretinal pigment epithelial fluid, the main manifestations of nAMD that drive treatment and treatment interval decisions.

Currently OCT machines are available only in the clinics. Therefore, the patients must be seen in the clinic to undergo an OCT imaging to help the physicians to determine if the disease has progressed and warrants treatment and to determine the proper interval for the next visit. For some elderly patients, continual frequent visits to clinics are advisable, since improved vision associated with anti-VEGF treatment may provide quality of life and economic value to patients and society [8]; however, this imposes significant burden on patients of transportation or caregiver availability, as well as the frequency of treatment that may lead to treatment fatigue and eventual discontinuation of treatment. This may hold especially true in the wake of COVID-19, as the typical AMD patient is at a higher risk for developing complications from the viral infection and may be more apprehensive about coming to the clinics for evaluation and treatments. It is, therefore, incumbent upon the treating retina physician community to find alternative ways to deliver high-quality care to ensure that patients do not lose vision while minimizing office visits.

An OCT home monitoring system that is able to detect the anatomic changes characteristic of nAMD would have the potential to reduce VA loss, the burden to patients, caregivers and clinics, and the costs of care related to nAMD. This system would overcome these pandemic and post-pandemic issues by personalizing the visits such that those who maintain anatomic stability may need to come in only when fluid reaccumulates, while those who need frequent injections based on OCT imaging will know that it is necessary to come in for injections. Home OCT may also support the management of patients undergoing longer acting treatments [9, 10] or the Port Delivery System (Genentech), which has been approved in the U.S. [11] The longer duration between the planned office visits and the variability in time to reactivation of nAMD could potentially benefit from a home monitoring system.

Adopting and enhancing OCT technology while maintaining an acceptable image quality and diagnostics efficacy can be challenging. Any home monitoring system should meet several key requirements:

1. The patient must be able to use the system properly and self-image without oversight from a trained technician.
2. The economics of the system should allow single patient use.
3. Given the large amount of data generated by frequently performed OCT self-imaging, images must be analyzed automatically to avoid overburdening the treating physician with review work.
4. An accompanying telemedicine infrastructure is necessary to securely transmit, store, analyze, and disseminate large amounts of personal health information; and
5. A dedicated remote diagnostic clinic infrastructure must be in place to support and monitor self-imaging compliance of patients who are using the system at home.

The Notal Vision Home OCT (NVHO) systems (Notal Vision Inc. Manassas, VA, USA), NVHO 2.5, a prototype and NVHO 3, the version to be commercialized, were developed to address several of those key requirements. The two models of the NVHO were different in their overall dimensions, however they share the same user interaction, key electro-optical specifications and scanning pattern. The purpose of the current studies was to validate the ability of self-imaging by patients with nAMD by comparing the performance of the NVHO in identifying intraretinal and subretinal fluid in eyes with nAMD to images acquired with commercial in-office OCT platforms. The study hypothesis is that the NVHO will meet the first two requirements for an OCT home monitoring system as listed above.

## Methods

Two clinical studies were performed with the NVHO. The first study with NVHO 2.5 was a prospective, cross-sectional study conducted from September 6, 2018 through February 18, 2019 at 4 retina care centers. The second study was with NVHO 3 conducted from December 2, 2019 through February 26, 2020, in two additional centers. The COVID-19 outbreak resulted in the NVHO 3 study being placed on hold prior to the scheduled completion, and accounts for the smaller number of patients enrolled in the NVHO 3 study.

The studies were registered at ClinicalTrials.gov (NCT03969303 [retrospectively registered; 31/05/2019] and NCT04078672 [prospectively registered 06/09/2019]; https://clinicaltrials.gov/ct2/show/NCT03969303 and https://clinicaltrials.gov/ct2/show/NCT04078672, respectively). The studies’ protocols were reviewed and approved by all applicable Institutional Review Boards (TLV Medical Center Helsinki Committee for NCT04078672 [reference study TLV 0339–19] and InteReview IRB for NCT04078672 [reference study CC2018.008]), and all subjects provided written informed consent to participate. All research was performed in accordance with the Declaration of Helsinki.

The two studies had significant similarities. These included the characteristics of the studies population, the studies setting in an office environment, the inclusion and exclusion criteria, the patients’ interaction method, the electro-optical design, the scanning pattern and the method of comparison to a reference commercial OCT.

Due to these similarities, this report includes the results from both studies. Inclusion criteria for both cohorts included adults with a previous diagnosis of nAMD in at least one eye who were able to undergo OCT imaging with both a commercial OCT device and the NVHO (either version). Eyes with Snellen VA of 20/400 or better were included. Each investigator could exclude a patient if they deemed the patient to have any condition that would prohibit participation.

### OCT scan devices

**Fig. 1:**
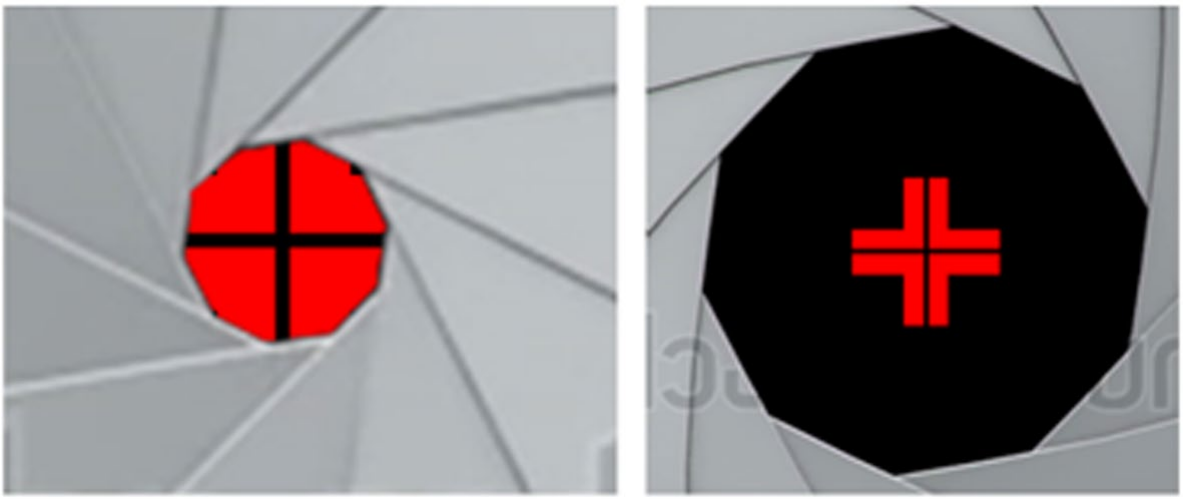
Forehead positioning feedback. Left image: Patient view with forehead not on forehead rest. Right image: Patient view with forehead gently touching the forehead rest

**Fig. 2:**
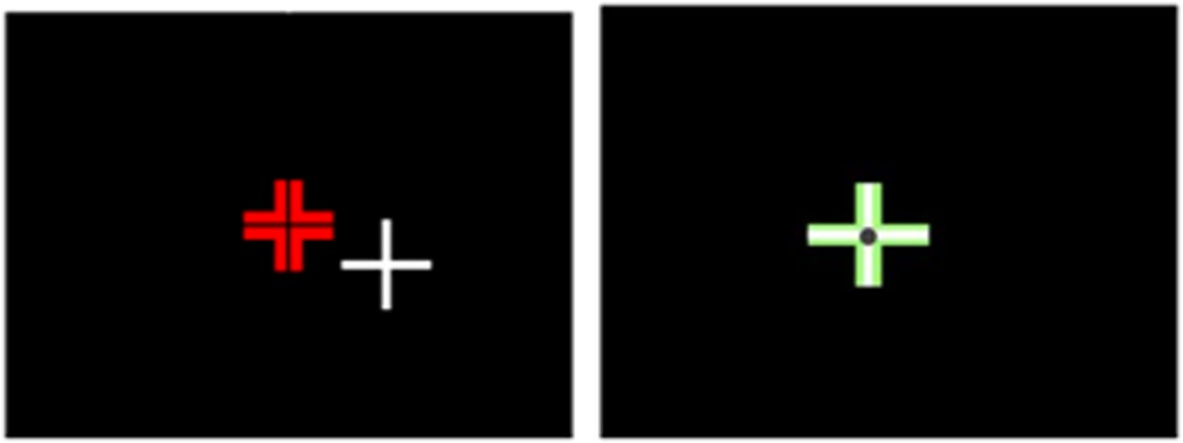
Visual X-Y direction head positioning and eye fixation feedback. Left image: Patient view requiring moving head laterally. Right image: Patient view when lateral alignment achieved

NVHO 2.5 is a device prototype and has a different overall shape than NVHO 3, the predominant difference being physical size with NVHO 2.5 weighing 45 Lb. and NVHO 3 weighing 16 Lb. The two models share the same patient controls, OCT electro-optical specifications, and user interaction enabling automatic self-imaging. Other similarities include patient ability to manually adjust the device height and to select which eye will be scanned. The patients self-positioning and fixation are guided by a proprietary, automatic visual feedback mechanism that instructs subjects to move their head first towards the device and then laterally, i.e. left-right and up-down, and once in proper position to look at a blinking fixation target for the duration of the scanning (Figs. 1 and 2), the image is taken.

Patients used either the NVHO 2.5 or NVHO 3 models in an ophthalmic clinic where the ophthalmic technician initiated the self-imaging process. Patients self-aligned their head and OCT scanning started automatically without technician intervention. Data was transferred from the device to a laptop for image processing, mimicking transmission to the cloud as planned when installed at patient’s homes. Both models used a pre-determined pattern of 88 B-scans across an area of 3 mm × 3 mm (10° × 10° field of view). The specification for both the NVHO 2.5 and NVHO 3 spectral-domain OCT includes a central wavelength of 830 nm, scan speed of 10,000 A-scans per second and 500 A-scans per B-scan, scan speed of 20 B-scans/sec, and total self-scanning time of approximately 40 seconds including patient self-realignments.

Commercial OCTs included the Cirrus-HD OCT (Carl Zeiss Meditec, Dublin, CA, USA) or the Spectralis OCT (Heidelberg Engineering, Heidelberg, Germany). Scanning setting of 128 × 512 A-scans were obtained with the Cirrus with a central wavelength of 840 nm, scan speed of 27,000–68,000 A-scans per second, 512 A-scans per B-scan, and total scanning time of approximately 3 seconds. Volume scans comprised of at least 40 B-scans were obtained with the Spectralis with a central wavelength of 870 nm scan speed of 85,000 A-scans per second and 768 A-scans per B-scan, and total scanning time that depends on the amount of eye movements. Both Cirrus and Spectralis scanned an area of 6 mm × 6 mm. The choice of commercial OCTs was determined by the platform available at each clinical center.

### Imaging procedures and interpretation

**Fig. 3:**
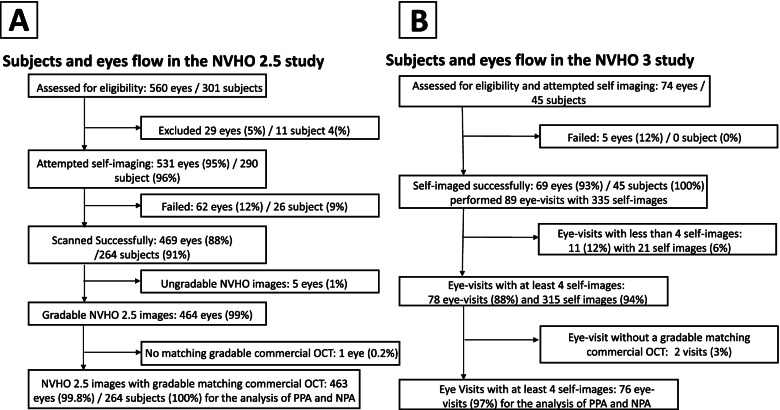
Flow diagrams illustrating the participants enrollment and exclusion throughout the studies

The ophthalmic exam during the screening visits included best-corrected VA (BCVA), verifying a diagnosis of nAMD, and identifying any comorbidities, including those that may preclude OCT imaging. A single volume scan of the study eye was obtained using the commercial OCT operated by trained clinical personnel. Enrolled subjects then underwent a 2-minute video tutorial and were subsequently tasked with placing their head in the proper location and fixating their better seeing eye on a target for the duration of the scan. During the subject’s first interaction, the device performs an auto-adjustment to personalize for the refractive error and eye length of a patient. Subjects using the NVHO 2.5 device performed a single self-imaging scan; subjects using the NVHO 3 performed a self-imaging practice scan and a session of up to four self-imaging scans to provide data on repeatability. After subjects obtained the self-imaging OCT scans, study personnel administered a subjective experience questionnaire to capture study subjects’ experience with the study device. All the NVHO and commercial OCT images were graded separately by a single ophthalmologist. To avoid possible bias in interpretation, the grading of the commercial OCT and NVHO images were performed separately without concurrent review of several volume scan. To minimize the possible bias due to grading by a single ophthalmologist, the grading was performed by the same grader for all the volume scans in the two reported studies, which include the NVHO and the reference images from commercial OCT with the intention that any personal bias will be reflected equally on the two sides of any comparison. Other than the presence or absence of intraretinal and subretinal fluid (IRF and SRF, respectively), the grader also indicated when fluid was present on the commercial OCT outside of the 3 mm × 3 mm image area. A flow diagrams illustrating the participants enrollment and exclusion throughout the studies is included in Fig. 3.

### Image analysis: manufacturer signal quality index (MSI)

The MSI is designed to provide the reviewing physician an automatically generated, objective, quantitative indication of image quality for clinical interpretation. The signal quality index for each B-scan (MSIB) is calculated based on retinal signal intensity and noise characteristics. The quality index for the entire volume scan (MSI) is based on the mean MSIB of all the eligible B-scans in the volume scan. The MSI and MSIB scale is from 0 (no visible retinal signal) to 7 (good). During the study of NVHO 2.5, for the purpose of validation, fifty B-scans were randomly selected and were manually graded for MSIB by two human graders, with the mean MSIB of the two graders serve as reference, (RefS MSIB), and later compared to the MSIB automatically calculated by the application. The MSI of volume scan from self-imaging with NVHO 3 were used to evaluate the repeatability of the self-imaging quality. The coefficient of variation (CV%) for each series was calculated. In addition, the distribution of the MSI for entire data set of NVHO 3 was calculated.

### Statistical analysis

The primary statistical goal of this study was to characterize the Positive Percent Agreement (PPA) and Negative Percent Agreement (NPA) of the two NVHO systems’ detection of intraretinal and subretinal fluid within the central 10° of the macula against the reference standard commercial OCT results as determined by a single ophthalmologist. The 95% confidence interval of the PPA and NPA were derived using the binomial distribution. It was determined that at least 100 eyes per analysis were required to provide a width of the 95% confidence interval of 0.1 assuming the true detection rate was 90%. A secondary analysis compared the fluid volume identified with the NVHO to the amount of fluid identified on the same eyes with the commercial OCT. The fluid volume quantification in nano liters (nl) was performed with the deep learning-based Notal OCT Analyzer (NOA), a validated tool [12–15] for analyzing commercial OCT and NVHO output. The results were presented in a scatter plot and Bland-Altman comparisons.

## Results

### Results of the NVHO 2.5 study

#### Demographics and disposition: NVHO 2.5

**Fig. 4:**
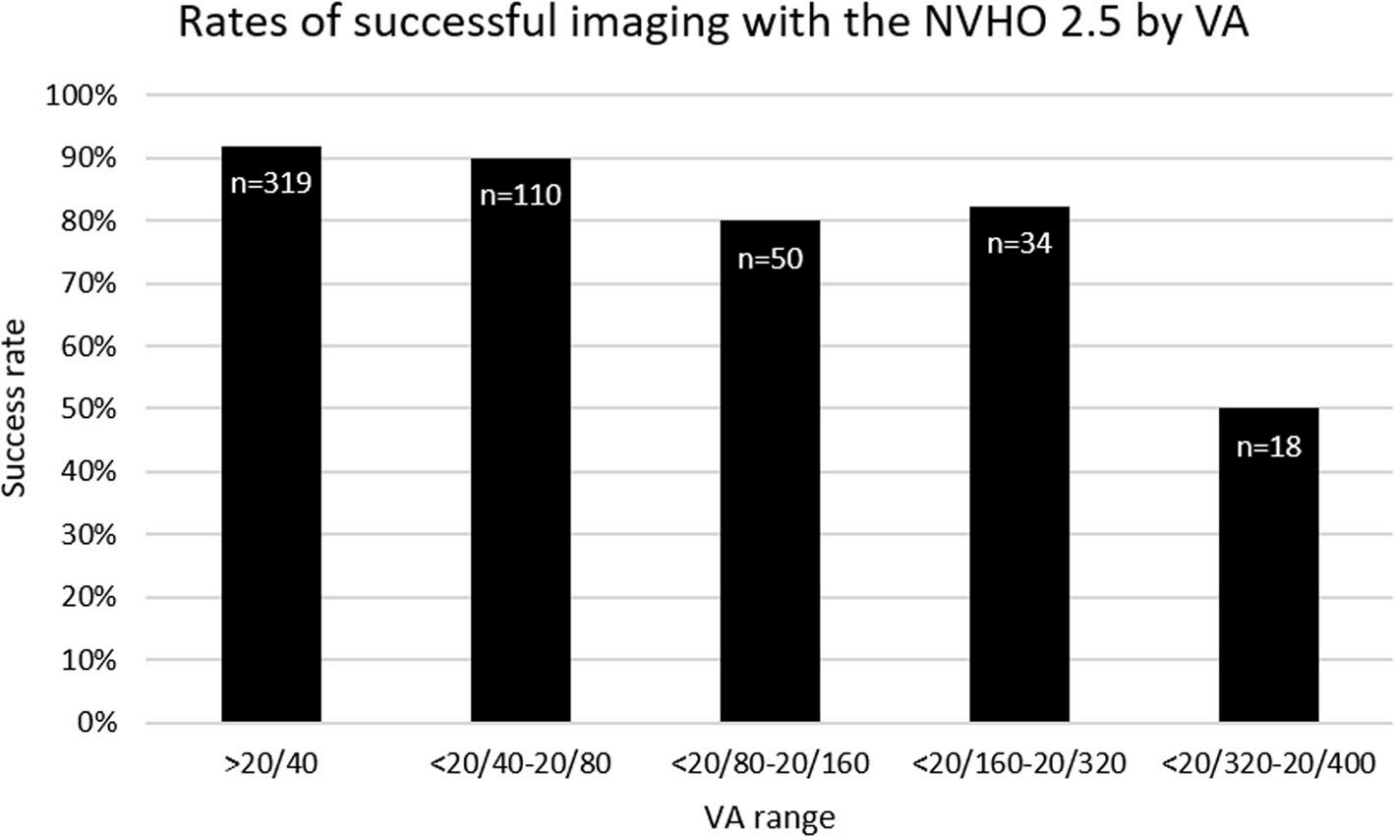
Rates of successful self-imaging with the NVHO 2.5 device by visual acuity

A total of 560 eyes of 301 subjects were enrolled to the NVHO 2.5 study. Eleven (4%) subjects were excluded, and 13 subjects continued with just one eye due to technical issues with the NVHO 2.5 OCT and had no usable images. Of the 531 eyes that attempted self-imaging, successful images were acquired in 469 (88%) of the eyes in 264 (91%) of the 290 subjects with the mean (SD) age of 78.8 (8.8); 153 (58%) were female with median VA of 20/40. Two hundred and thirty-one (80%) of the 290 subjects were in the U.S. and 59 in Israel. Comparison of the patients and eyes that succeeded or failed in self-imaging including the distribution of disease characteristics and VA are shown in Table 1. Most eyes that were able to be successfully self-imaged had VA better than 20/40 and were diagnosed with nAMD at enrollment. Subjects’ ability to successfully acquire an image was dependent upon VA (Fig. 4) with a minimum VA of 20/320 needed for success. Eyes with VA above this threshold consistently demonstrated 80–90% success, while eyes with VA below this threshold were successful less than half the time. Sixty-two of the 531 eyes failed self-imaging, 22 (36%) with mean (range) visual acuity of 20/78 (20/25–20/400) failed both eyes of 11 subjects, 25 (40%) eyes with mean (range) visual acuity of 20/83 (20/20–20/400) failed while the fellow eye was able to complete self-imaging and 15 (24%) eyes with mean (range) visual acuity of 20/62 (20/25–20/400) were single eyes that attempted and did not complete self-imaging. The reference commercial OCT images were Cirrus in 83% and Spectralis in 17%.

**Table 1:** Baseline characteristics by ability to complete self-imaging

	Completed self-imaging NVHO 2.5	Could not complete self-imaging NVHO 2.5	Total / *p*-value	Completed self-imaging NVHO 3	Could not complete self-imaging NVHO 3	Total / *p*-value
N (%) - patients	264 (91)	26 (9)	290	45	0	
N (%) - eyes	469 (88)	62 (12)	531	69	5	74
Age (SD)	78.8 (8.8)	84.3 (7.8)	< 0.001	79.5 (6.5)	84.0 (1.4)	0.14
Gender, n (%) female	153 (58)	15 (56)		23 (51)	2, (40)	
LogMAR VA, Mean (SD)	0.35 (0.30)	0.58 (0.41)	< 0.001	0.29 (0.30)	0.47 (0.25)	0.2
Mean VA Snellen equivalent	20/45	20/76		20/39	20/59	
LogMAR VA, Median (IQR)	0.30 (0.2,0.48)	0.52 (0.22,0.88)		0.18 (0.10,0.40)	0.52 (0.40–0.7)	
Median VA Snellen equivalent	20/40	20/66		20/30	20/66	
**Visual acuity, n (%)**						
≥20/40	293 (62)	26 (42)		48 (69.6)	1 (20)	
< 20/40–20/80	99 (21)	11 (18)		14 (20.9)	2 (40)	
< 20/80–20/160	40 (9)	10 (16)		0 (0)	2 (40)	
< 20/160–20/320	28 (6)	6 (10)		7 (10.1)	–	
< 20/320–20/400	9 (2)	9 (15)		0 (0)	–	
**Diagnosis, n (%)**^a^						
Early AMD	38 (8.1)			6 (8.7)		
Intermediate AMD	121 (25.8)	–		16 (23.2)		
Neovascular AMD	310 (66.1)			47 (68.1)		

*VA* Visual acuity, *SD* Standard deviation, *IQR* Interquartile range, *AMD* Age-related macular degeneration

^a^Information of diagnosis of eye that did not complete self-imaging was not collected in full

#### Positive percent agreement and negative percent agreement of NVHO 2.5

**Fig. 5:**
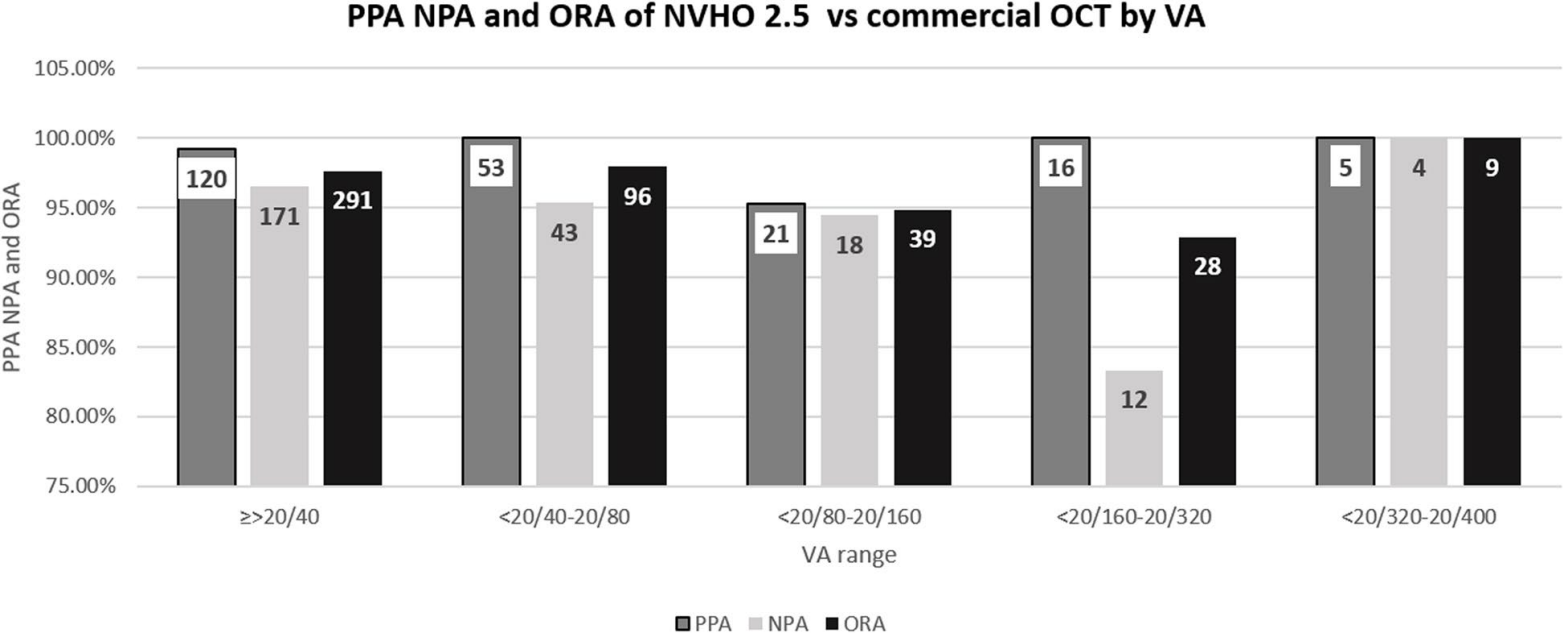
Positive Precent Agreement (PPA), Negative Precent Agreement (NPA) and Overall Rates of Agreement (ORA) of NVHO 2.5 device versus commercial OCT by visual acuity

Of the 469 eyes with image acquisition using the NVHO 2.5, 464 (99%) were deemed gradable by the reading ophthalmologist. Likewise, 468/469 (99.8%) images acquired by commercial OCT were deemed gradable. 463 eyes of 264 subjects had gradable images both from the commercial and the NVHO 2.5 OCTs and were available for grading and comparison of the presence or absence of fluid (Fig. 3A). For the NVHO 2.5 device, the Positive Percent Agreement (PPA) and Negative Percent Agreement (NPA) for detecting the presence of any fluid (SRF and/or IRF), SRF, and IRF were 98%/96, 93%/96, and 91%/98%, respectively (Table 2). This diagnostic accuracy was not dependent on VA, with agreement rates (PPA and NPA and ORA) of 0.83–1.0 across all VA levels (Fig. 5).

**Table 2:** Positive Percent Agreement (PPA) and Negative Percent Agreement (NPA) of NVHO 2.5 OCT for detecting subretinal and intraretinal fluid versus commercial OCT imaging

Notal Box	Commercial OCT			PPA and NPA
2.5	Fluid	No Fluid	Total	(95% CI per binomial distribution)
**Presence of Fluid**				
Fluid	213	9	222	PPA: 213/217 = 98% (95.3, 99.5%)
No Fluid	4	237	241	NPA: 237/246 = 96% (93.2, 98.3%)
Total	217	246	463	
**Subretinal fluid**				
Fluid	152	13	165	PPA: 152/163 = 93% (88.2, 96.6%)
No Fluid	11	287	298	NPA: 287/300 = 96% (92.7, 97.7%)
Total	163	300	463	
**Intraretinal fluid**				
Fluid	89	9	98	PPA: 89/98 = 91% (83.3, 95.7%)
No Fluid	9	356	365	NPA: 356/365 = 98% (95.4, 98.9%)
Total	98	365	463

#### Presence of fluid outside the central 10° × 10° field of view

Among the 469 eyes, there were three eyes (0.6%) with fluid observed on the commercial OCT outside of the scanned field of the NVHO 2.5 and with no fluid within the central 10°.

#### Comparison of fluid volume

**Fig. 6:**
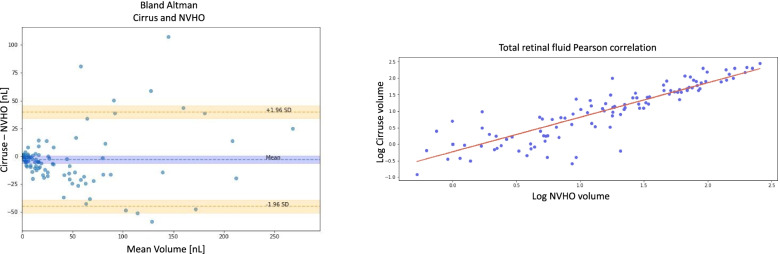
Comparison of the fluid volume identified on Cirrus vs the NVHO V2.5

Of the 257 eyes imaged with Cirrus OCT and paired self-image with the NVHO V2.5, 118 eyes had fluid identified on Cirrus by a human grader and the NOA was able to analyze. The fluid volume range was 0.12 to 280.6 nl for the Cirrus and 0 to 256.0 nl for the NVHO 2.5. The mean fluid volume (SD) was 33.9 (54.0) nl and 36.6 (51.1) nl for the Cirrus and NVHO 2.5 respectively (*p* = 0.35). The Pearson Correlation was 0.916 with a slop of 0.967. A Bland-Altman analysis resulted in a mean (95% CI) difference of − 2.7 (− 6,0.6) nl, upper Limit of Agreement (+ 1.96 SD, 95% CI)) of 39.7 (34.0,45.5) nl and lower Limit of Agreement (− 1.96 SD, 95%CI) of − 45.2 (− 51.0,-36.5) nl (Fig. 6).

### Subjective experience with NVHO 2.5

Subjects in the U.S. (*n* = 231) using the NVHO 2.5 were given a patient questionnaire consisting of 10 questions on user experience. Of the 146 respondents 96% indicated “strongly agree” or “agree” with statements on the simplicity and comfort of the NVHO 2.5 (Additional file 1: Appendix A).

### Results of the NVHO 3 study

#### Demographics, disposition and testing performance: NVHO 3

A total of 45 subjects (74 eyes) were enrolled in the NVHO 3 study with the mean (SD) age of 79.5 (6.5) years, 23 (51%) female, and median VA of 20/30. The majority of eyes that were able to successfully self-image had a VA better than 20/40 and were diagnosed with nAMD at enrollment. Of these, 45 subjects (100%) and 69 eyes (93%) completed the self-imaging with NVHO 3 while five eyes of five subjects were not able to successfully complete self-imaging. Of the five subjects that were unable to perform a self-image, one subject could not keep one eye open (VA: 20/50), one subject could not see the crosses on the internal screen of the NVHO V3 with one of their eyes (VA:20/100), and three subjects could not follow the fixation target with one of their eyes (VA in those eyes were: 20/66, 20/100, and 20/21;). Details and the distribution of the diagnosis and VA at enrollment are shown in Table 1. The volume scans acquired by commercial OCT during the study were with Cirrus in 65% and Spectralis in 35%.

**Fig. 7:**
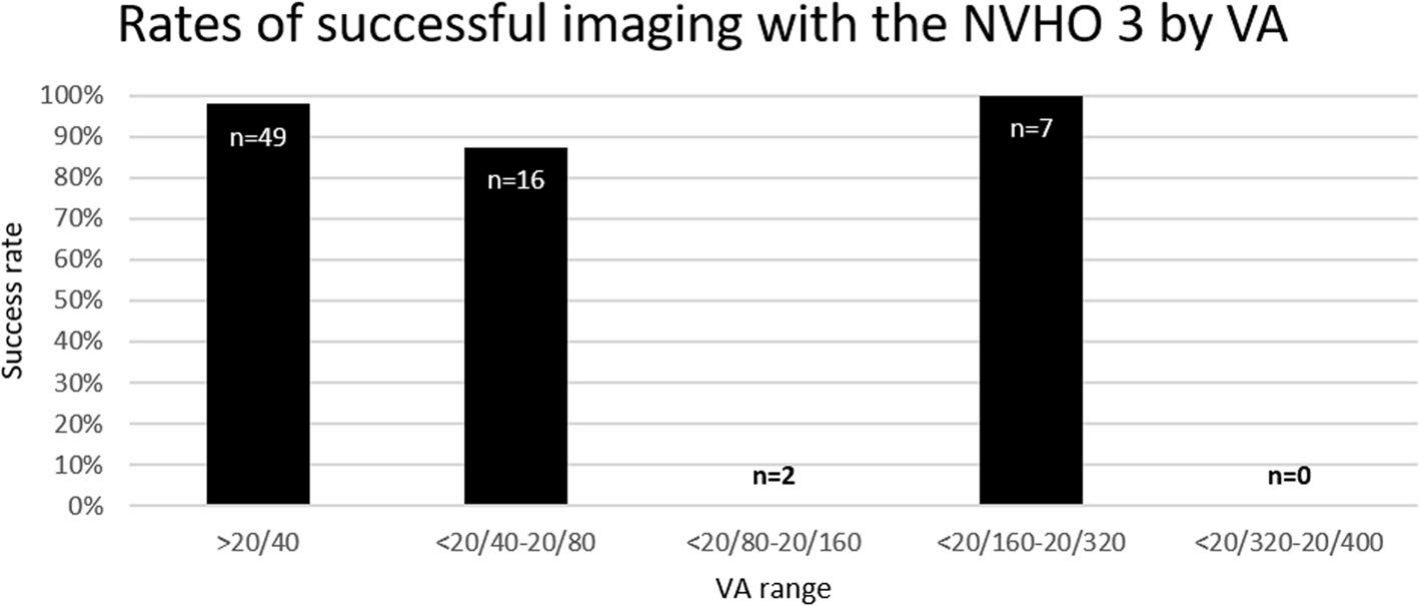
Rates of successful imaging with the NVHO 3 device by visual acuity

In contrast to the NVHO 2.5 study in which there was a single self-imaging visit, during the NVHO 3 study, the subjects arrived at the clinic several times and performed several self-imaging sessions. For the purposes of this analysis, each visit where subjects performed between one to five self-imaging sessions was defined as an “eye visit.” A total of 336 self-images were performed by 45 subjects with 69 eyes over 89 eye visits. Although there was variability in the number of visits and number of self-imaging, during most (87.7%) of the eye visits, at least four self-images were performed, accounting for 315 (93.4%) of the total 336 self-images, which allow enough data for analysis (Fig. 3B). Subjects’ ability to successfully acquire an image was not dependent upon VA (Fig. 7), however that finding is limited due to the lack of information about eyes with VA worse than 20/320. The distribution of the 336 self-images over the eye-visits is shown in Table 3. The mean (SD) imaging duration for the 336 self-images was 42 (31) seconds with a median (interquartile range, IQR) of 32 (25,46) seconds. Among the 78 eye-visits of 62 eyes that had at least four repeated self-images, the median imaging duration for each of the first four self-images were median (IQR) 31 (25,41), 31 (27,43), 29 (25,40) and 33 (25,46) respectively.

**Table 3:** Disposition of the eyes over the NVHO 3 eye-visits

No. of Self-Images during a visit	Eye visits, n (%)	Self-Image count
1	5 (5.6%)	5
2	2 (2.2%)	4
3	4 (4.5%)	12
4	75 (84.3%)	300
5	3 (3.4%)	15
Total	89 (100%)	336

#### Positive percent agreement and negative percent agreement of NVHO 3

**Fig. 8:**
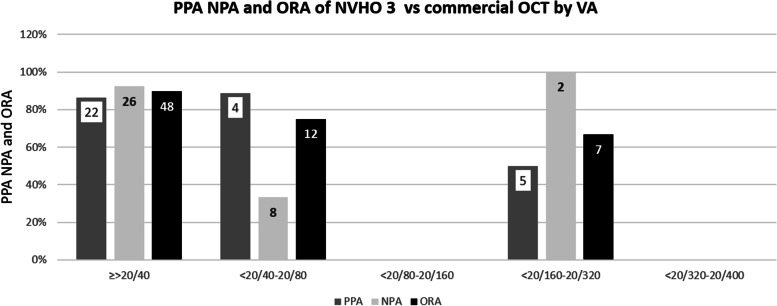
Positive Precent Agreement (PPA), Negative Precent Agreement (NPA) and Overall Rates of Agreement (ORA) of NVHO 3 device versus commercial OCT by visual acuity

**Fig. 9:**
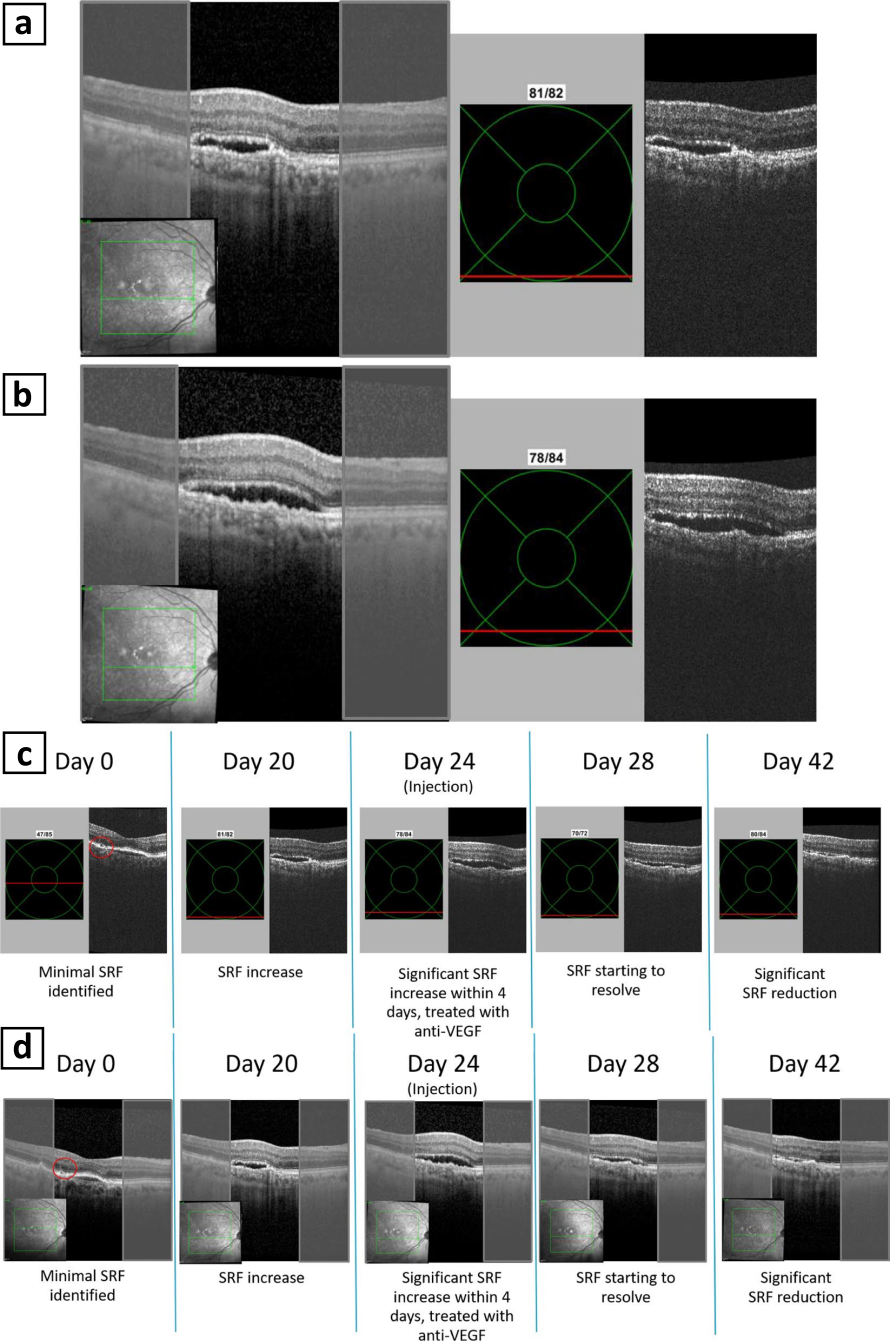
**a** Left: Spectralis OCT image; Right: NVHO 3 image. **b** Left: Spectralis OCT image; Right: NVHO 3 image. **c** An example of comparison of a series of scans over 42 days – NVHO 3 images. **d** An example of comparison of a series of scans over 42 days – Spectralis images

During the NVHO 3 study, 62 eyes during 78 eye-visits had four sequential self-images in a single visit, defined as a practice self-image and three gradable self-images. Of these, during 76 eye-visits a corresponding commercial OCT scan was performed while two subjects declined to remain in the office for a commercial OCT scan. All gradable self-images and the commercial OCT were graded by an in-house ophthalmologist for presence or absence of fluid. The 76 eyes-visits analyzed for PPA and NPA included data from one eye-visit of 54 eyes, from two eye-visits of one eye, from three eye-visits of two eyes, from four eye-visits of one eye and from five eye-visits of 2 eyes. For the NVHO 3 device, the PPA and NPA for detecting the presence of any fluid (subretinal and/or intraretinal), subretinal fluid, and intraretinal fluid were defined as the identification of fluid in at least one of the three NVHO repeated self-images. The PPA/NPA were 97%/95, 96%/94 and 100%/98%, respectively. Table 4 includes the PPA/NPA data while reviewing, the first, the first two and the three volume scans and identifying fluid if it was observed in at least one of the volume scans. This diagnostic accuracy was not dependent on VA, with accuracy rates (average of PPA and NPA) of 0.71 to 0.94 across all VA levels (Fig. 8). Examples of side-by-side representation of fluid with the NVHO 3 and a commercial OCT and an example of comparison of a series of scans over 42 days are shown in Figs. 9a-d.

**Table 4:** Presence and absence of retinal fluid, based on number of NVHO 3 self-images reviewed

NVHO	Commercial OCT			PPA and NPA
	+	–	Total	(95% CI)
**Fluid status was defined as the identification of fluid in the first NVHO 3 repeated self-image**				
+	34	1	35	PPA: 34/38 = 89% (75, 97%)
–	4	37	41	NPA: 37/38 = 97% (86, 100%)
Total	38	38	76	
**Fluid status was defined as the identification of fluid in at least one of the two NVHO 3 repeated self-images**				
+	36	2	38	PPA: 36/38 = 95% (82, 99%)
–	2	36	38	NPA: 36/38 = 95% (82, 99%)
Total	38	38	76	
**Fluid status was defined as the identification of fluid in at least one of the three NVHO 3 repeated self-images**				
+	37	2	39	PPA: 37/38 = 97% (86, 100%)
–	1	36	37	NPA: 36/38 = 95% (82, 99%)
Total	38	38	76	
**SRF status was defined as the identification of SRF fluid in the first NVHO 3 repeated self-image**				
+	25	2	27	PPA: 25/27 = 93% (76, 99%)
–	2	47	49	NPA: 47/49 = 96% (86, 100%)
Total	27	49	76	
**SRF status was defined as the identification of SRF fluid in at least one of the two NVHO 3 repeated self-images**				
+	26	3	29	PPA: 26/27 = 96% (81, 100%)
–	1	46	47	NPA: 46/49 = 94% (83, 99%)
Total	27	49	76	
**SRF status was defined as the identification of SRF fluid in at least one of the three NVHO 3 repeated self-images**				
+	26	3	29	PPA: 26/27 = 96% (81, 100%)
–	1	46	47	NPA: 46/49 = 94% (83, 99%)
Total	27	49	76	
**IRF status was defined as the identification of IRF fluid in the first NVHO 3 repeated self-image**				
+	15	1	16	PPA: 15/17 = 88% (64, 99%)
–	2	58	60	NPA: 58/59 = 98% (91, 100%)
Total	17	59	76	
**IRF status was defined as the identification of IRF fluid in at least one of the two NVHO 3 repeated self-images**				
+	16	1	17	PPA: 16/17 = 94% (71, 100%)
–	1	58	59	NPA: 58/59 = 98% (91, 100%)
Total	17	59	76	
**IRF status was defined as the identification of IRF fluid in at least one of the three NVHO 3 repeated self-images**				
+	17	1	18	
–	0	58	58	PPA: 17/17 = 100% (80, 100%)
Total	17	59	76	NPA: 58/59 = 98% (91, 100%)

*SRF* Subretinal fluid, *IRF* Intraretinal fluid

#### Presence of fluid outside the central 10° × 10° field of view

Among the 69 eyes, there was one eye (1.4%) with fluid observed on the commercial OCT outside of the scanned field of the NVHO 3 and with no fluid within the central 10°.

#### Results from NVHO 2.5 and NVHO 3: validation of the MSI

To validate the MSIB, we compared the automatically generated MSIB set to those MSIB that were manually and independently graded by two human graders (RefS MSIB) in 50 B-scans from self-imaging with NVHO 2.5. Table 5 shows that the agreement between the automatically generated MSIB and the RefS MSIB is better than the agreement between the two graders. As the MSI is the mean value of all the MSIB in the volume scan, its validation from the validation of MSIB is straightforward. For each series of four self-images of the 78 eye-visits with NVHO 3, total of 312 volume scans and the mean and standard deviation (SD) of the four MSIs was calculated. The overall Mean CV% for that entire dataset was 12.4%, and the mean MSI for the four self-images were 3.5, 3.5, 3.6, and 3.5 respectively.

**Table 5:** Comparison of the agreement between the MSIB and the Reference Standard MSIB (RefS MSIB) to the agreement between the two graders, by cumulative distribution of difference in agreement as well as by Bias and SD

Window of differences in MSIB	MSIB prediction vs RefS MSIB	Grader 1 vs Grader 2
	Percentage of B-scans within the window	
0	42%	42%
≤ ± 1	88%	76%
≤ ± 2	96%	92%
≤ ±3	100%	100%
≤ ±4	100%	100%
≤ ±5	100%	100%
Bias (mean of differences)	−0.18	0.42
SD of differences	1.063	1.247

#### Subjective experience with NVHO 3

Subjects using the NVHO 3 were given a patient questionnaire consisting of 10 questions on user experience. There were 37 total respondents (82%) to the questionnaire (the remainder were not included due to administration errors and in one case, because of a language barrier). Of the respondents, 91% “strongly agree” or “agree” with statements on the simplicity and comfort of the NVHO V3.0 (Additional file 2: Appendix B). The remaining participants were either not asked to answer the questionnaire (administrative error) or could not fill it out due to language issues.

## Discussion

Results of these two studies with the NVHO system showed the requirements of acceptable image quality to allow retinal fluid identification through patient self-imaging can be met in a high number of eyes: 88% of eyes imaged with the NVHO 2.5 and 93% of eyes imaged with the NVHO 3 successfully self-imaged at least ones. Patients that could not image with any of the models were older and the ones that failed self-imaging had worse VA. There was no significant relationship between the status of the fellow eye and the ability to self-image. The images were successfully self-captured solely by patients moving their head and gaze in response to directional visual feedback. This is in contrast to clinic-based OCT systems requiring the technician to move the imaging head while needing the patient to hold their head steady.

Both versions of the NVHO were intuitive and easy to operate by an elderly patient population with impaired vision, evidenced by high rate of subjects being able to capture gradable images, even when only video instructions were given. It appears that the device ergonomics assisted the self-imaging process well.

The NVHO showed a high PPA/NPA for identifying the presence (within the central 10° of the macula) of any fluid (subretinal and/or intraretinal) when compared to a commercial OCT. The tight 34 μm spacing between B-scans supports the intended use of NVHO as a retinal fluid finder.

In order to allow a higher level of comparison between the NVHO and commercial OCT, an AI-based analysis method was used to quantify and compare the biomarker of fluid volume of pairs of eyes imaged with the Cirrus and the NVHO 2.5. The NOA was utilized, and the result show high correlation (CC = 0.916) between the 2 imaging systems, with close proximity to the 45° line. A Bland-Altman resulted in a minimal bias of − 2.7 (− 6,0.6) nl and Limits of Agreements (LoA) of approximately 40 nl with observed dependency on the absolute fluid volume, i.e. a smaller LoA for smaller volumes.

The accessibility of the system in a home setting allows multiple volume scans to be obtained in a daily or close to daily frequency of self-imaging, which in turn may reduce the risk of missing recurrence of retinal fluid. The study showed that for NVHO 3, the review of up to three volume scans increased the PPA of identification of any fluid from 89 to 97% and decreased the NPA of identification of fluid from 97 to 95%. Similar trends were observed for SRF and IRF alone, however, these findings are inconclusive due to the small number of patients included in the NVHO 3 study.

The MSI was validated against human graders and was consistent during repeat testing, which validated the system’s automated imaging capabilities. The self-reported patients experience with device and tutorial were very positive and should support patient compliance with daily self-imaging.

The device’s diagnostic performance generated few false-positives that would prompt unnecessary additional office visits and fewer false-negatives so that true worsening (as indicated by fluid accumulation detected by commercial OCT) was rarely missed by the device with approximately 1% of commercial OCT scans showing fluid exclusively outside the 10° field imaged by the NVHO. Thus, the NVHO device may be useful to monitor between visits for patients with nAMD, and also for patients with intermediate AMD to detect early conversion to nAMD before central VA is affected. The latter indication is particularly relevant for patients receiving intravitreal anti-VEGF injections that have dry AMD in the fellow eye deemed at high risk of conversion.

The strengths of this study include comparison to the current clinical standard — commercial OCT, generation of evidence with state of the art AI-based tools for quantification of the biomarker of fluid volume, as well as the inclusion of subjects with impaired central acuity in the study eye. The NVHO 3 showed the same outcomes as the NVHO 2.5, thereby alleviating the typical concerns about using prototype devices in clinical studies.

Maloca et al. studied the safety and feasibility of a sparse OCT device prototype for patient-delivered retina home monitoring [16]. The device met patient ergonomic requirements but was limited in its imaging capabilities. A small number of B-scans was used to measure the retinal thickness. Also, they did not perform quantitative side-by-side comparison of the accuracy to detect retinal fluid. Similarly, von der Burchard et al. studied and reported pilot results with a different technology of full-field OCT, demonstrating the ability of subjects with a variety of retinal conditions to perform a self-imaging task at a range of ages and visual acuities, however the report was limited to general ability to identify related biomarkers while pointing at certain image quality limitations and without attempting to get at any statistical conclusions about the imaging performance of these relevant biomarkers [17]. The NVHO 2.5 and NVHO 3 studies were limited to several aspects of self-imaging enabling home OCT, including that enrolling the intended number of study subjects for the NVHO 3 version was hindered by the COVID-19 pandemic and resulted in fewer than the intended sample size. However, those who were enrolled provided enough images to allow for statistical analysis. Intergrader conflicts were nonexistent as only one ophthalmologist graded all images. Other limitations included the use of ophthalmic clinic setting while reporting self-imaging success rates, and that majority of eyes had visual acuity better than 20/40 which could have positively affected the results. Further studies will be required to evaluate the performance of a complete home OCT system.

In summary, it seems that there is a general unmet need and interest in a home OCT monitoring solution which is being challenged by a growing number of companies. One of these systems is the subject matter of this report. The investigated patient self-operated SD-OCT systems meet several key design requirements for remote home monitoring of patients with nAMD. More than 90% of the enrolled subjects with nAMD were able to obtain OCT images of their own disease-affected and the fellow eyes, in most incidences central 10° field imaged captured the fluid status of interest, and the self-operated OCT device image quality compared favorably to the commercial OCT, suggesting that this planned device may be able to complement standard-of-care clinical assessments and treatments.

## Supplementary Information

**Additional file 1.**

**Additional file 2.**

## Data Availability

The data that support the findings of this study are available from Notal Vision, but restrictions apply to the availability of these data, which were used under license for the current study, and so are not publicly available. Any data requests should be addressed to gidi@notalvision.com.

## Abbreviations

BCVA: Best-corrected visual acuity
MSI: Manufacturer signal quality index
MSIB: Manufacturer signal quality index B-scan
nAMD: Neovascular age-related macular degeneration
NPA: Negative percent agreement
NVHO: Notal Vision HOME OCT
OCT: Optical coherence tomography
PPA: Positive percent agreement
SD-OCT: Spectral-domain optical coherence tomography
VA: Visual acuity
VEGF: Vascular endothelial growth factor
