# Supplementary material for: Evaluation of a self-imaging SD-OCT system designed for remote home monitoring
Source: BMC Ophthalmol. 2022 Jun 10;22:261. doi: 10.1186/s12886-022-02458-z (PMC9186475; doi:10.1186/s12886-022-02458-z)
Supplement: Supplementary file 1 — Additional file 1. [file 12886_2022_2458_MOESM1_ESM.pdf]

Appendix A: Subjects' subjective experience with V2.5

	Strongly agree	Agree	Uncertain	Disagree	Strongly disagree
The demonstration (movie) was helpful	65%	33%	1%	1%	0%
The tutorial session was clear	69%	29%	2%	0%	0%
I understand the tasks I must do to scan my eye	62%	32%	4%	1%	1%
The tasks I had to do to scan my eye were easy to perform	64%	33%	2%	1%	1%
Resting between the sessions helped me to complete the test	44%	43%	9%	3%	0%
Testing duration was short	66%	31%	0%	1%	1%
I felt comfortable during the test (posture, head rest)	67%	31%	0%	1%	1%
I didn't feel that my eyes are getting tired or burning during the test	64%	33%	1%	1%	0%
The viewer's mask was comfortable while performing the test	65%	32%	2%	0%	1%
The handles of the device were helpful to position myself	56%	36%	9%	0%	0%
