# Supplementary material for: Evaluation of a self-imaging SD-OCT system designed for remote home monitoring
Source: BMC Ophthalmol. 2022 Jun 10;22:261. doi: 10.1186/s12886-022-02458-z (PMC9186475; doi:10.1186/s12886-022-02458-z)
Supplement: Supplementary file 2 — Additional file 2. [file 12886_2022_2458_MOESM2_ESM.pdf]

Appendix B: Subjects' subjective experience with V3

	Strongly agree	Agree	Uncertain	Disagree	Strongly disagree
The demonstration (movie) was helpful	54%	35%	3%	8%	0%
The tutorial session was clear	65%	35%	0%	0%	0%
I understand the tasks I must do to scan my eye	57%	35%	8%	0%	0%
The tasks I had to do to scan my eye were easy to perform	49%	46%	5%	0%	0%
Resting between the sessions helped me to complete the test	41%	46%	14%	0%	0%
Testing duration was short	57%	35%	8%	0%	0%
I felt comfortable during the test (posture, head rest)	57%	30%	5%	5%	3%
I didn't feel that my eyes are getting tired or burning during the test	57%	32%	5%	5%	0%
The viewer's mask was comfortable while performing the test	62%	27%	3%	8%	0%
The handles of the device were helpful to position myself	62%	30%	3%	5%	0%
